# Natural course of unruptured intracranial aneurysms: a case surveillance study in China

**DOI:** 10.3389/fneur.2025.1566246

**Published:** 2025-04-30

**Authors:** Xiuhu An, Linchun Huan, Pengran Liu, Yan Zhao, Nai Zhang, Yaohua Li, Yunpeng Lin, Jiwen Wang, Jiheng Hao, Xinyu Yang, Bangyue Wang

**Affiliations:** ^1^Department of Neurosurgery, Tianjin Medical University General Hospital, Tianjin, China; ^2^Department of Neurosurgery, Linyi People’s Hospital, Linyi, Shandong, China; ^3^Department of Neurosurgery, Liaocheng People’s Hospital, Liaocheng, Shandong, China; ^4^Department of Neurosurgery, The Second Affiliated Hospital of Anhui Medical University, Hefei, China

**Keywords:** intracranial aneurysm, natural course, current status, risk factors, rupture rate

## Abstract

**Background:**

The natural course of unruptured intracranial aneurysms (UIAs) has been well described in developed countries, but there is a lack of large studies on UIAs in China. This article aims to fill this gap by detailing the current status and natural course of UIAs in China and identifying the major risk factors for their rupture, providing a basis for clinical decision-making.

**Methods:**

We included all patients with UIAs consecutively admitted to 12 tertiary care centers in 4 provinces in northern China between January 2017 and December 2020. The mean follow-up was 3.1 years (range 0–7.3 years). The current status of UIA patients in China was described in detail. Risk ratios for rupture were analyzed using the Cox proportional hazards model, and Kaplan–Meier curves were analyzed for long-term rupture rates.

**Results:**

In this study, among the 1,475 patients, 33.4% declined surgical treatment. Of the 1,189 patients who completed follow-up, 10.3% initially received conservative treatment but later underwent surgery. A total of 1,337 patients with UIAs who met the criteria were included in the statistical analysis. The annual rupture rate was 1.75%. Cox proportional hazards model identified the following risk factors for rupture: age over 70 years (HR 2.136, 95% CI 1.302–3.504, *p* = 0.003), aneurysm size of 10–20 mm (HR 3.543, 95% CI 1.501–8.363, *p* = 0.004) and ≥20 mm (HR 4.455, 95% CI 1.034–19.187, *p* = 0.045). ICA (HR 0.427, 95% CI 0.203–0.897, *p* = 0.025) was a protective factor.

**Conclusion:**

In China, treatment options for UIA patients are unique, with a low willingness to undergo surgery leading to a higher rupture rate. A significant percentage of Chinese patients refuse treatment, and those who initially choose conservative management are unlikely to opt for surgical intervention later. Advanced age, specific locations, and size are associated with UIA rupture. This study has important implications for clinical decision-making, public awareness of UIAs, and the development of health policies.

## Introduction

Cerebrovascular disease is one of the leading causes of death in China, contributing to a significant disease burden (1). The widespread use of neurovascular imaging has increased the detection of unruptured intracranial aneurysms (UIAs) (2). Studies indicate that the prevalence of UIAs in the Chinese population aged 35–75 years is as high as 7% (3). Although the lifetime risk of UIA rupture is low, with an annual rupture rate of approximately 1% (2, 4, 5), subarachnoid hemorrhage from a ruptured aneurysm remains catastrophic, with a mortality rate of 35–50% (6, 7). Since the 1970s, surgical intervention has been employed to prevent aneurysm rupture, even when the risk is relatively low (8). Between 65 and 85% of UIAs are smaller than 5–7 mm in diameter and have a lower rupture risk (4). While surgery can reduce rupture risk, it also increases the financial burden on patients and strains healthcare resources (9). As a result, a large proportion of the UIA population in China remains untreated for various reasons.

China is a large country with a large population, and a reasonable distribution of medical resources is crucial. A rationale for which populations of UIAs require surgical intervention is needed. Although the natural history and annual rupture rates of UIAs have previously been reported in detail by ISUIA and UCAS, there is a lack of large-scale studies on the natural history of UIAs in China (10–12). To fill this gap, we conducted a multicenter cohort study of UIAs in China to describe the current status and natural history of UIAs in China and to identify risk factors for rupture to guide clinical decision making.

## Methods

### Design and population

In this retrospective cohort study, we included all consecutive patients with UIAs admitted to 12 tertiary care centers across 4 provinces in northern China between January 2017 and December 2020. The data for the study were obtained from the China Cerebral Aneurysm Database (CMAD). This is a multi-center, retrospective, observational database registered in China. As an observational study, only routine clinical data were collected, and the data were de-identified. Therefore, informed consent was not required, and the study was approved by the regulatory authorities. The study design was approved by the Ethics Committee of the General Hospital of Tianjin Medical University (IRB2022-YX-175-01), and registration was completed on the China Clinical Trial Registry website under the clinical trial number (ChiCTR2200065083) (13). All analyses were performed in accordance with the Declaration of Helsinki and local ethics guidelines.

Inclusion criteria: (1) Age ≥ 18 years; (2) Patients were diagnosed with UIAs by at least one of CTA, MRA, DSA; (3) Intracranial aneurysm morphology was saccular; (4) They did not undergo surgical treatments during hospitalization at the relevant medical centers, including endovascular treatment (EVT) and microsurgical treatment (MST).

Exclusion criteria: (1) fungal aneurysms, entrapment aneurysms, coarctation aneurysms, or traumatic aneurysms; (2) patients with intracranial aneurysms; (3) patients with comorbid arteriovenous malformations, arteriovenous fistulas, or moyamoya disease; and (4) patients who underwent surgical procedures during follow-up, including EVT and MST.

### Measurements

Retrospective data were obtained from standardized electronic medical records. We collected the following information: age (≤70 or >70 years), sex, residence (urban or rural), history of smoking, history of alcohol consumption, history of hypertension, history of diabetes mellitus, history of previous stroke (hemorrhagic or ischemic), location of aneurysm [middle cerebral artery (MCA), cavernous part of the internal carotid artery (ICA), internal carotid artery (ICA), anterior communicating artery (AComA), anterior cerebral artery (ACA), posterior communicating artery (PComA), basilar tip and basilar–superior cerebellar artery, vertebral artery–posterior inferior cerebellar artery and vertebrobasilar junction], size of aneurysm (<5, 5–7, 7–10, 10–20, and ≥20 mm), and number of aneurysms (single or multiple). The size of the aneurysm was measured from CTA, MRA, and DSA imaging data, and the aneurysm size was defined as the maximum diameter.

### Follow-up

Follow-up assessments were conducted between 2022 and 2024 through standardized telephone interviews with patients or their relatives. Verbal consent was obtained from the patient or family during the follow-up visit. Missed visits were defined as failures to contact the patient or family on three or more occasions. When an aneurysm ruptured, the patients or their relatives sought medical attention, and they were informed of the aneurysm rupture diagnosis during the clinical care process. This information was obtained from the patients or their relatives during the follow-up interviews. Follow-up data were collected from the time of UIA detection to rupture, including the patient’s condition, follow-up treatment, and time to rupture if it occurred. Survival time was defined as the interval between UIA detection and rupture. Data collection for each patient was terminated when the aneurysm ruptured, the patient died, or no further follow-up was possible.

After reviewing the medical records in the database and validating them through follow-up, reasons for conservative management were recorded and categorized as follows: (1) UIA too small for surgical indication (too small); (2) refusal of treatment; (3) severe condition on admission; (4) anticipated surgical challenge (too risky); (5) referral to another hospital (referral); and (6) unclear. We classified (1) as not requiring surgery, and (2), (3), (4), and (5) as requiring surgery but not treated.

### Statistical analysis

Continuous variables were expressed as mean ± standard deviation or median (IQR); categorical variables were expressed as frequencies (percentages). Continuous variables were compared using the non-parametric Mann–Whitney *U* test. Comparisons of categorical variables were performed using the Pearson chi-squared test or the Fisher exact test.

A multiple imputation approach was used to address the missing values. The full conditional specification method was used to estimate the missing values of the variables using the iterative Markov Chain Monte Carlo (MCMC) method. The MCMC method generates multiple possible imputed datasets, simulating and estimating the distribution of missing values, thereby reducing the bias that may arise from using a single imputation method (14). Multifactor analysis was applied to the data after multiple imputation.

Risk ratios for rupture were analyzed using the Cox proportional hazards model. All variables underwent univariate analysis, and those with *p* < 0.05 were subsequently included in the multivariate analysis. Kaplan–Meier curves were used to assess long-term rupture rates. Patients were censored for loss to follow-up, with the follow-up time for censored events defined as the interval between UIA detection and censoring.

Statistical analysis was performed with SPSS version 25 (IBM Corp, Armonk, New York) and R version 4.2.3 (R Foundation for Statistical Computing, Vienna, Austria). A two-tailed *p*-value of less than 0.05 was considered statistically significant.

## Results

**Figure 1:**
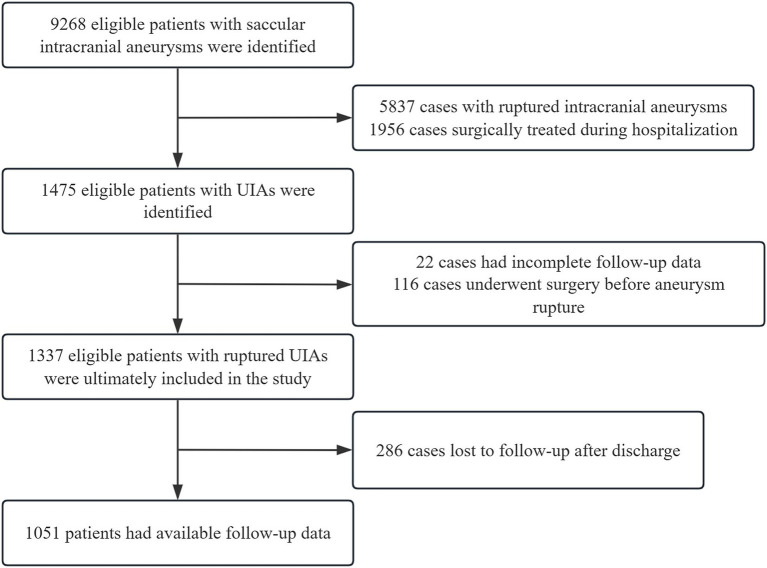
Detailed flow chart of the study population from CMAD.

Between 2017 and 2020, we included 1,475 patients with UIAs from 12 tertiary care centers. Of these, 22 cases with incomplete follow-up data and 116 cases treated surgically before rupture were excluded. A total of 1,337 patients with UIAs who met the criteria were included in the analysis. The median age was 63 years (IQR 55.0–69.0), and 758 (56.7%) were female. A detailed flow chart of the study population from CMAD is shown in Figure 1. A history of previous stroke was present in 306 (22.9%) cases. Of the aneurysms, 148 cases (11.3%) were located in the MCA, 110 cases (8.4%) in the cavernous part of the internal carotid artery, 519 cases (39.6%) in the ICA, 76 cases (5.8%) in the AComA, 54 cases (4.1%) in the ACA, 273 cases (20.9%) in the PComA, 91 cases (7.0%) in the basilar tip and basilar–superior cerebellar artery, and 38 cases (2.9%) in the vertebral artery–posterior inferior cerebellar artery and vertebrobasilar junction. The median size of the largest aneurysm was 3.5 mm (IQR 2.5–5.0 mm), with 735 (75.2%) patients having an aneurysm size <5 mm, 129 (13.2%) patients with an aneurysm size of 5–7 mm, 55 (5.6%) patients with 7–10 mm, 40 (4.1%) patients with 10–20 mm, and 18 (1.8%) patients with ≥20 mm. Baseline characteristics before and after multiple imputation are presented in Table 1 and Supplementary Table S1.

**Table 1:** Baseline characteristics before multiple imputation.

Baseline characteristics		Total, *N*
Age, y, median (IQR)	63 (55.0–69.0)	1,337
≤70	1,082 (80.9)	
>70	255 (19.1)	
Sex, *n* (%)		1,337
Male	579 (43.3)	
Female	758 (56.7)	
Residence area, *n* (%)		1,337
Rural	847 (63.4)	
Urban	490 (36.6)	
Lifestyle risk factors, *n* (%)		
Smoking	237 (17.7)	1,336
Alcohol	158 (11.8)	1,334
Medical history, *n* (%)		
Hypertension	810 (60.6)	1,336
Diabetes	175 (13.1)	1,336
Previous stroke	306 (22.9)	1,337
Location of the largest aneurysm, *n* (%)		1,309
MCA	148 (11.3)	
Cavernous part of carotid artery	110 (8.4)	
ICA	519 (39.6)	
AComA	76 (5.8)	
ACA	54 (4.1)	
PComA	273 (20.9)	
Basilar tip and basilar–superior cerebellar artery	91 (7.0)	
Vertebral artery–posterior inferior cerebellar artery and vertebrobasilar junction	38 (2.9)	
Size of the largest aneurysm, mm, *n* (%)		977
Median (IQR)	3.5 (2.5–5.0)	
<5	735 (75.2)	
5–7	129 (13.2)	
7–10	55 (5.6)	
10–20	40 (4.1)	
≥20	18 (1.8)	
Number with unruptured aneurysms, *n* (%)		1,337
Single	1,086 (81.2)	
Multiple	251 (18.8)	

ICA, internal carotid artery; AComA, anterior communicating artery; ACA, anterior cerebral artery; MCA, middle cerebral artery; PComA, posterior communicating artery.

Among the 1,475 patients, the most common reason for conservative treatment was that the UIA was too small with no indication for surgery (51.7%), followed by refusal of surgical treatment (33.4%). Of the 1,189 patients who completed follow-up, only 10.3% of patients initially treated conservatively subsequently underwent surgery. Among these patients, the reason for conservative treatment was refusal of surgical treatment in 9.6% of cases (Table 2 and Supplementary Table S1).

**Table 2:** The reasons for conservative treatment at discharge in 1,475 patients with unruptured intracranial aneurysms.

Reason	Patients	
	*N*	%
Too small	762	51.7
Refusal of treatment	492	33.4
Severe condition	74	5.0
Too risky	61	4.1
Referral	45	3.1
Unclear	41	2.8

### Natural course of the aneurysms

**Figure 2:**
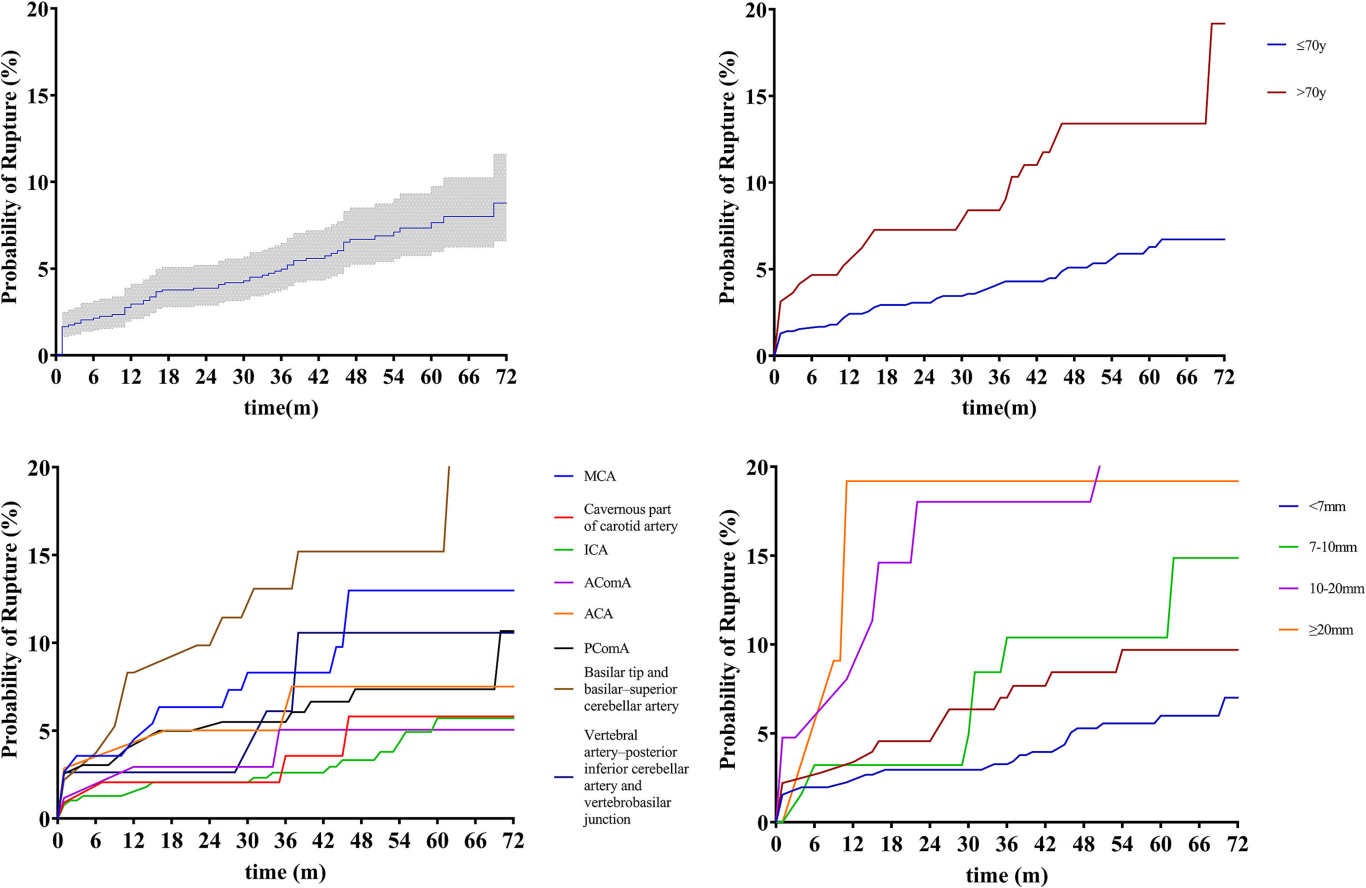
Kaplan–Meier curves assessing aneurysm rupture during follow-up.

Figure 2 shows Kaplan–Meier curves assessing aneurysm rupture during follow-up. The mean follow-up period was 3.1 years (range 0–7.3 years). During the 4,110 person-years of follow-up, there were 72 rupture events, resulting in an annual rupture rate of 1.75%.

A total of 571 patients who did not require surgery and 448 patients who required surgery were included in the survival analysis. The annual rupture rate of aneurysms that required surgery but were not treated was 1.94%, slightly higher than that of those not requiring surgery (1.39%), but the difference was not statistically significant (*p* = 0.17) (Supplementary Figure S1).

Univariable Cox proportional hazards modeling identified several risk factors for rupture: age over 70 years (HR 2.467, 95% CI 1.525–3.992, *p* < 0.001), history of hypertension (HR 2.013, 95% CI 1.181–3.432, *p* = 0.010), history of previous stroke (HR 1.873, 95% CI 1.147–3.059, *p* = 0.012), multiple aneurysms (HR 1.923, 95% CI 1.156–3.197, *p* = 0.012), and aneurysm size of 10–20 mm (HR 4.699, 95% CI 2.103–10.500, *p* < 0.001). ICA (HR 0.347, 95% CI 0.168–0.714, *p* = 0.004) was identified as a protective factor. Multivariable analysis confirmed that age over 70 years (HR 2.136, 95% CI 1.302–3.504, *p* = 0.003), aneurysm size of 10–20 mm (HR 3.543, 95% CI 1.501–8.363, *p* = 0.004) and aneurysm size ≥20 mm (HR 4.455, 95% CI 1.034–19.187, *p* = 0.045) was significant risk factors for rupture. ICA (HR 0.427, 95% CI 0.203–0.897, *p* = 0.025) was a protective factor. The results of the Cox proportional hazards model are shown in Table 3.

**Table 3:** Risk factors associated with rupture of cerebral aneurysms.

Risk factors	Univariable analysis		Multivariable analysis	
	HR (95% CI)	*P-*value	HR (95% CI)	*P*-value
Age				
≤70	Reference		Reference	
>70	2.467(1.525–3.992)	<0.001	2.136(1.302–3.504)	0.003
Sex				
Male	Reference		NA	NA
Female	1.083(0.676–1.735)	0.739	NA	NA
Residence area				
Rural	Reference		NA	NA
Urban	0.724(0.436–1.204)	0.213	NA	NA
Lifestyle risk factors				
Smoking	0.960(0.516–1.784)	0.896	NA	NA
Alcohol	0.980(0.470–2.044)	0.957	NA	NA
Medical history				
Hypertension	2.013(1.181–3.432)	0.010	1.429(0.821–2.487)	0.206
Diabetes	1.161(0.611–2.207)	0.648	NA	NA
Previous stroke	1.873(1.147–3.059)	0.012	1.619(0.974–2.691)	0.063
Location of the largest aneurysm				
MCA	Reference		Reference	
Cavernous part of carotid artery	0.397(0.130–1.219)	0.107	0.420(0.133–1.323)	0.138
ICA	0.347(0.168–0.714)	0.004	0.427(0.203–0.897)	0.025
AComA	0.427(0.122–1.499)	0.184	0.524(0.147–1.863)	0.318
ACA	0.680(0.222–2.086)	0.500	0.816(0.261–2.549)	0.726
PComA	0.660(0.320–1.359)	0.260	0.716(0.342–1.500)	0.376
Basilar tip and basilar–superior cerebellar artery	1.465(0.656–3.271)	0.351	1.285(0.568–2.903)	0.547
Vertebral artery–posterior inferior cerebellar artery and vertebrobasilar junction	0.856(0.244–3.004)	0.808	0.680(0.188–2.462)	0.557
Number with unruptured aneurysms				
Single	Reference		Reference	
Multiple	1.923(1.156–3.197)	0.012	1.562(0.922–2.646)	0.097
Size of the largest aneurysm, mm				
<5	Reference		Reference	
5–7	1.702(0.953–3.039)	0.072	1.195(0.650–2.196)	0.566
7–10	2.040(0.914–4.553)	0.082	1.287(0.534–3.102)	0.574
10–20	4.699(2.103–10.500)	<0.001	3.543(1.501–8.363)	0.004
≥20	3.520(0.850–14.577)	0.083	4.455(1.034–19.187)	0.045

## Discussion

Our results show that the overall annual incidence of ruptured UIAs from 2017 to 2024 is 1.75%. We found that UIA rupture was influenced by advanced age, specific aneurysm location, and size. Additionally, the most common reason for conservative management was refusal of surgical treatment (33.4%), followed by lack of surgical indication for UIAs (51.7%). Only 10.3% of patients who initially refused surgical treatment were subsequently treated surgically. To the best of our knowledge, this study is the largest and most comprehensive with the longest follow-up among studies conducted in China, involving data from 12 hospitals in northern China. It aims to systematically characterize the natural history of UIAs in China and identify risk factors for rupture. Our findings have important implications for understanding UIAs and for clinical decision-making.

Although previous studies have detailed the natural history of UIAs, most have been conducted in developed countries. This study is the first to report the annual rupture rate of UIAs in China (1.75%), which is higher than the rates reported in developed countries (0.5–1.3%) (3–5, 12, 15). One possible reason for this difference is that 42.9% of patients with UIAs in our study were treated conservatively, a much higher proportion compared to the 33.6% reported by Alshekhlee et al. (16). We found that the annual rupture rate of aneurysms that required surgery but were not treated was 1.94%, higher than that of those not requiring surgery (1.39%), although the difference was not statistically significant. The reason for conservative treatment in as high as 33.4% of UIA patients in this study was their refusal to undergo surgical treatment. During follow-up, only 10.3% of our cohort underwent surgical treatment, significantly lower than the 45.5% in the UCAS study (12). We analyzed that financial burden was a major factor contributing to the refusal of surgery (17). High medical costs can be a significant barrier, particularly in the absence of adequate health insurance support. Although the Chinese population generally has medical insurance coverage, the subsidies are low, making it difficult for some families to afford the deposits required during hospitalization (18). Qureshi et al. (19) reported that the overall mortality rate of UIAs ranges from 1.36 to 6.30%. A randomized trial demonstrated that the 1-year morbidity and mortality rates were 4.2 and 3.6%, respectively, in patients undergoing MST and EVT (20). The ISUIA study reported that the 30-day overall morbidity and mortality rates were 13.7% for MST and 9.3% for EVT (10). Additionally, a meta-analysis found that among studies on clipping of unruptured aneurysms, the postoperative mortality rate was 2.6%, while the morbidity rate was 10.9% (21). As a result, some families weigh the cost of surgery against the potential benefits of post-operative care. Beyond economic challenges, other factors such as complex family backgrounds, low levels of formal education, and mistrust of healthcare professionals may also contribute to these decisions. Consequently, there is a need for improvements in the healthcare system in northern Chinese cities.

It has been shown that the risk of UIA rupture is closely related to their specific location in the cerebrovascular system (10, 22, 23). Consistent with the available evidence, we found that compared to MCA, ICA reduced the rupture risk by 57.3%. This is in line with the findings of the UCAS study (12). We hypothesize that this increased risk may be related to several factors. The internal carotid artery has a relatively tough vessel wall and is generally more stable, with lower blood flow velocity. The structure of the ICA vessel wall (such as thicker adventitia and intima) may provide better resistance to stretching, thereby reducing the rupture risk of aneurysms. ICA typically experiences lower blood pressure and relatively stable blood flow velocity, making aneurysms formed in this area less susceptible to high-flow impacts. Interestingly, the UCAS study showed that aneurysms most likely to rupture were located in the anterior and posterior communicating arteries, whereas posterior circulation aneurysms (other than those in the posterior communicating arteries and larger aneurysms) were not found to be at higher risk of rupture (12). However, this phenomenon was not observed in our study. Therefore, larger multicenter studies are needed to further elucidate the rupture risk associated with specific locations of UIAs.

The risk of rupture for larger UIAs is well-documented. The intracranial aneurysm growth risk score (ELAPSS) indicates that the risk of aneurysm rupture increases with size (24). A large clinical study of 6,697 intracranial aneurysms found that the rupture rate for aneurysms with a maximum diameter of 7–9 mm was 3.35%, for those with a diameter of 10–24 mm was 9.09%, and for aneurysms >25 mm was as high as 76.26% (12). Similarly, we found that compared to UIAs <5 mm, the rupture risk of UIAs sized 10–20 mm and ≥20 mm increased by 154.3 and 345.5%, respectively. These findings suggest that surgical treatment is recommended for aneurysms ≥10 mm to mitigate the risk of rupture and its potentially severe consequences.

Age has been identified as a risk factor for UIA rupture, though both positive and negative effects have been reported (15, 22, 25). Previous studies indicate that the incidence of subarachnoid hemorrhage due to UIA rupture increases with age, rising from approximately 1.5–2.5 per 100,000 people per year in the third decade of life to about 40–78 per 100,000 people per year in the eighth decade (26, 27). Our study found that patients with UIAs over 70 years of age had a 113.6% increased risk of rupture compared to those 70 years or younger. This finding is consistent with the results reported by Tominari et al. (28). The higher incidence of complications in elderly patients is often due to reduced physical reserves, and studies have shown that elderly patients with complications tend to have worse outcomes than those without (29). Therefore, it is crucial for the healthcare team to develop a treatment plan for older patients with UIAs that carefully balances risks and benefits, with the goal of maximizing the patient’s quality of life and health outcomes.

### Limitation

This study has several limitations. First, as an observational study, it cannot establish causality between risk factors and aneurysm rupture. Future prospective studies are needed to explore whether these associations are causal. Second, while our data were collected from 12 tertiary care centers in northern China, the study’s hospital-based dataset does not represent the entire population of China. A nationally representative sample is needed for more comprehensive future research. Third, the lack of follow-up imaging data prevented us from visualizing changes in aneurysm size over time. Fourth, although we made efforts to minimize the loss to follow-up, 20.1% of patients still lacked follow-up data. However, there were no significant differences in baseline characteristics between the lost to follow-up group and the completed follow-up group (Supplementary Table S3). Finally, we did not compare the rupture rates between treated and untreated aneurysms. It would have been more meaningful if we could determine the rupture rate among patients who should have been treated but were not.

## Conclusion

In China, treatment options for UIA patients present unique challenges. A notable reluctance to undergo surgery among the Chinese UIA population contributes to a high rupture rate. Specifically, a significant percentage of Chinese patients refuse treatment, and once conservative management is chosen, the likelihood of subsequent surgical intervention remains low. Advanced age, specific locations, and size are associated with UIA rupture. These findings have important implications for clinical decision-making, public awareness of UIAs, and health policy development.

## Data Availability

The raw data supporting the conclusions of this article will be made available by the authors, without undue reservation.
