# Supplementary material for: Natural course of unruptured intracranial aneurysms: a case surveillance study in China
Source: Front Neurol. 2025 Apr 30;16:1566246. doi: 10.3389/fneur.2025.1566246 (PMC12075362; doi:10.3389/fneur.2025.1566246)

Table S1. Baseline characteristics after multiple imputation.

| Baseline Characteristics |  | Total, N |
| --- | --- | --- |
| Age, y, median (IQR) | 63 (55.0-69.0) | 1337 |
| ≤70 | 1082 (80.9) |  |
| >70 | 255 (19.1) |  |
| Sex, n (%) |  | 1337 |
| Male | 579 (43.3) |  |
| Female | 758 (56.7) |  |
| Residence area, n (%) |  | 1337 |
| Rural | 847 (63.4) |  |
| Urban | 490 (36.6) |  |
| Lifestyle risk factors, n (%) |  |  |
| Smoking | 237 (17.7) | 1337 |
| Alcohol | 160 (12.0) | 1337 |
| Medical history, n (%) |  |  |
| Hypertension | 811 (60.7) | 1337 |
| Diabetes | 175 (13.1) | 1337 |
| Previous stroke | 306 (22.9) | 1337 |
| Location of the largest aneurysm, n (%) |  | 1337 |
| MCA | 151 (11.3) |  |
| Cavernous part of carotid artery | 110 (8.2) |  |
| ICA | 519 (38.8) |  |
| AComA | 85 (6.4) |  |
| ACA | 70 (5.2) |  |
| PComA | 273 (20.4) |  |
| Basilar tip and basilar–superior cerebellar artery | 91 (6.8) |  |
| Vertebral artery–posterior inferior cerebellar artery and vertebrobasilar junction | 38 (2.8) |  |
| Size of the largest aneurysm, mm, n (%) |  | 1337 |
| Median (IQR) | 3.8 (2.8-5.2) |  |
| <5 | 964 (72.1) |  |
| 5-7 | 227 (17.0) |  |
| 7-10 | 86 (6.4) |  |
| 10-20 | 42 (3.1) |  |
| ≥20 | 18 (1.3) |  |
| Number with unruptured aneurysms, n (%) |  | 1337 |
| Single | 1086 (81.2) |  |
| Multiple | 251 (18.8) |  |

Table S2. Follow-up treatment and management of 1,189 untreated unruptured aneurysms.

|  | Total  N=1189 | Too small  N=631 | Refusal of treatment  N=350 | Severe condition  N=61 | Too risky  N=42 | Referral  N=37 | Unclear  N=31 |
| --- | --- | --- | --- | --- | --- | --- | --- |
| Untreated | 1067  (89.7) | 582  (92.2) | 350  (90.4) | 53  (86.9) | 29  (69.0) | 27  (73.0) | 26  (83.9) |
| Treated | 122  (10.3) | 49  (7.8) | 37  (9.6) | 8  (13.3) | 13  (31.0) | 10  (27.0) | 5  (16.1) |

Table S3. Comparison of baseline characteristics between the lost and included populations.

| Baseline Characteristics | Missing  N=286 | Present  N=1051 | P value |
| --- | --- | --- | --- |
| Age, y, median (IQR) |  |  |  |
| ≤70 | 242 (84.6) | 840 (79.9) | 0.073 |
| >70 | 44 (15.4) | 211 (20.1) |  |
| Sex, n (%) |  |  | 0.878 |
| Male | 125 (43.7) | 454 (43.2) |  |
| Female | 161 (56.3) | 597 (56.8) |  |
| Residence area, n (%) |  |  | 0.204 |
| Rural | 172 (60.1) | 675 (64.2) |  |
| Urban | 114 (39.9) | 376 (35.8) |  |
| Lifestyle risk factors, n (%) |  |  |  |
| Smoking | 51 (17.8) | 186 (17.7) | 0.958 |
| Alcohol | 41 (14.3) | 119 (11.3) | 0.164 |
| Medical history, n (%) |  |  |  |
| Hypertension | 168 (58.7) | 643 (61.2) | 0.454 |
| Diabetes | 27 (9.4) | 148 (14.1) | 0.039 |
| Previous stroke | 70 (24.5) | 236 (22.5) | 0.471 |
| Location of the largest aneurysm, n (%) |  |  | 0.628 |
| MCA | 32 (11.2) | 119 (11.3) |  |
| Cavernous part of carotid artery | 21 (7.3) | 89 (8.5) |  |
| ICA | 113 (39.5) | 406 (38.6) |  |
| AComA | 25 (8.7) | 60 (5.7) |  |
| ACA | 17 (5.9) | 53 (5.0) |  |
| PComA | 54 (18.9) | 219 (20.8) |  |
| Basilar tip and basilar–superior cerebellar artery | 18 (6.3) | 73 (6.9) |  |
| Vertebral artery–posterior inferior cerebellar artery and vertebrobasilar junction | 6 (2.1) | 32 (3.0) |  |
| Size of the largest aneurysm, mm, n (%) |  |  | 0.955 |
| Median (IQR) |  |  |  |
| <5 | 207 (72.4) | 757 (72.0) |  |
| 5-7 | 47 (16.4) | 180 (17.1) |  |
| 7-10 | 19 (6.6) | 67 (6.4) |  |
| 10-20 | 8 (2.8) | 34 (3.2) |  |
| ≥20 | 5 (1.7) | 13 (1.2) |  |
| Number with unruptured aneurysms, n (%) |  |  | 0.015 |
| Single | 218 (76.2) | 868 (82.6) |  |
| Multiple | 68 (23.8) | 183 (17.4) |  |

Figure S1. The Kaplan-Meier curve for patients who required surgery versus those who did not require surgery.


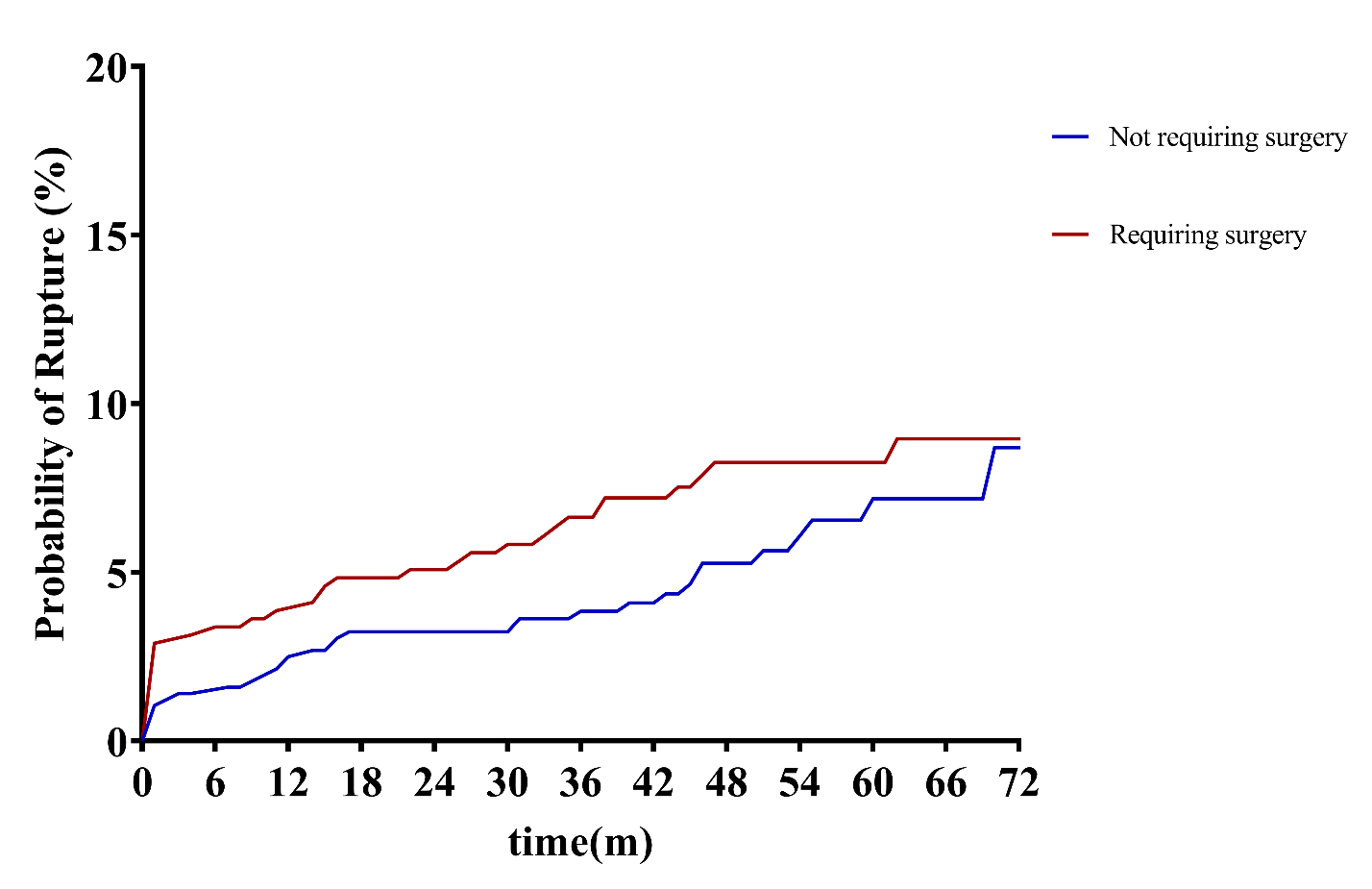

Supplement: Supplementary file 1 [file Table_1.docx]
